# Enhancing Locomotor Learning With Transcutaneous Spinal Electrical Stimulation and Somatosensory Augmentation: A Pilot Randomized Controlled Trial in Older Adults

**DOI:** 10.3389/fnagi.2022.837467

**Published:** 2022-03-02

**Authors:** David J. Clark, Kelly A. Hawkins, Steven P. Winesett, Brigette A. Cox, Sarah Pesquera, Jon W. Miles, David D. Fuller, Emily J. Fox

**Affiliations:** ^1^Brain Rehabilitation Research Center, Malcom Randall VA Medical Center, Gainesville, FL, United States; ^2^Department of Aging and Geriatric Research, University of Florida, Gainesville, FL, United States; ^3^Department of Physical Therapy, University of Florida, Gainesville, FL, United States; ^4^Department of Applied Physiology and Kinesiology, University of Florida, Gainesville, FL, United States; ^5^Brooks Rehabilitation, Jacksonville, FL, United States

**Keywords:** aging, older adults, walking, locomotion, direct current stimulation, brain, spinal cord, somatosensory

## Abstract

This study investigated locomotor learning of a complex terrain walking task in older adults, when combined with two adjuvant interventions: transcutaneous spinal direct current stimulation (tsDCS) to increase lumbar spinal cord excitability, and textured shoe insoles to increase somatosensory feedback to the spinal cord. The spinal cord has a crucial contribution to control of walking, and is a novel therapeutic target for rehabilitation of older adults. The complex terrain task involved walking a 10-meter course consisting of nine obstacles and three sections of compliant (soft) walking surface. Twenty-three participants were randomly assigned to one of the following groups: sham tsDCS and smooth insoles (*sham/smooth*; control group), sham tsDCS and textured insoles (*sham/textured*), active tsDCS and smooth insoles (*active/smooth*), and active tsDCS and textured insoles (*active/textured*). The first objective was to assess the feasibility, tolerability, and safety of the interventions. The second objective was to assess preliminary efficacy for increasing locomotor learning, as defined by retention of gains in walking speed between a baseline visit of task practice, and a subsequent follow-up visit. Variability of the center of mass while walking over the course was also evaluated. The change in executive control of walking (prefrontal cortical activity) between the baseline and follow-up visits was measured with functional near infrared spectroscopy. The study results demonstrated feasibility based on enrollment and retention of participants, tolerability based on self-report, and safety based on absence of adverse events. Preliminary efficacy was supported based on trends showing larger gains in walking speed and more pronounced reductions in mediolateral center of mass variability at the follow-up visit in the groups randomized to active tsDCS or textured insoles. These data justify future larger studies to further assess dosing and efficacy of these intervention approaches. In conclusion, rehabilitation interventions that target spinal control of walking present a potential opportunity for enhancing walking function in older adults.

## Introduction

Physical rehabilitation is the most effective approach for restoring or preserving walking function in older adults (Buford et al., 2014). However, there is a continued need to further augment the effects of rehabilitation and to understand why some people experience less functional benefit than others. Aging related degradation of the structure and function of the central and peripheral nervous systems may be an important factor. Previous research in this area has focused on changes in brain structure/activity, muscle activity, and somatosensation. In contrast, age-related changes of the spinal cord are under-investigated. These changes include structural degeneration (Nagashima and Oota, 1974; Tomlinson and Irving, 1977), slower and reduced neuronal excitation (Morita et al., 1995; Scaglioni et al., 2002; Kido et al., 2004), and increased neural noise (Brooke et al., 1989; Faisal et al., 2008; Hong and Rebec, 2012; Doidge, 2015).

The spinal cord plays a crucial role in control of walking. The lumbar region of the spinal cord contains complex circuits of the locomotor central pattern generator (CPG), which is thought to control patterned muscle activation for intra- and inter-limb coordination (Dietz, 2003; McCrea and Rybak, 2008; Gosgnach et al., 2017). The CPG may contribute to “automaticity” of steady state walking, thereby reducing the need for executive control (i.e., cognitive effort) to coordinate walking (Clark, 2015). Somatosensory information is an important source of input to the CPG (Frigon and Rossignol, 2006; Frigon, 2017), and provides real time feedback that might modulate CPG timing of gait characteristics (Guertin et al., 1995; Dietz, 1996; Hiebert et al., 1996; Pearson, 2008). Somatosensory information also elicits reflex responses to unexpected perturbations (e.g., striking an obstacle) (Zehr and Stein, 1999). During complex tasks such as walking over uneven terrain or stepping over an obstacle, the spinal cord integrates descending motor commands with ongoing CPG activity to transiently adjust gait mechanics (Michel et al., 2007; Haefeli et al., 2011). Rehabilitation interventions that target spinal control of walking present a potential opportunity for enhancing walking function in older adults. The use of complex walking tasks in rehabilitation may be particularly beneficial due to their applicability to real-world walking in the home and community (Shumway-Cook et al., 2003), as well as their ability to strongly engage control networks at multiple levels of the neuraxis.

A cornerstone of physical rehabilitation is motor learning. This study investigated learning of a complex terrain walking task when combined with two adjuvant interventions targeting spinal control: transcutaneous spinal direct current stimulation (tsDCS) and textured shoe insoles. tsDCS is a non-invasive neuromodulation approach that applies a weak electrical current to the spinal cord via electrodes placed on the skin. The electrical current is intended to modify neuron cell membrane polarization, thereby affecting the probability of eliciting action potentials within spinal circuits (Bocci et al., 2014, 2015). tsDCS has shown promise in modulating spinal reflex activity and contributing to gains in physical performance, but has not been assessed in the context of augmenting locomotor learning in older adults. Similarly, textured shoe insoles may enhance spinal excitation by increasing somatosensory afferent input during walking. Prior studies have demonstrated performance gains and/or changes in neural activity when older adults walk in textured shoe insoles (Palluel et al., 2008; Qiu et al., 2012; Clark et al., 2014). When combined with a behavioral task (e.g., a complex walking task), both tsDCS and textured insoles may promote task-specific activation and enhance Hebbian neuroplasticity (“fire together wire together”) in spinal networks that contribute to locomotor learning and performance.

Therefore, the long term objective of this line of research is to test whether intervention adjuvants that target spinal circuits may improve locomotor learning of a complex terrain walking task. For this particular study, the first goal was to assess the feasibility (participant enrollment and drop-out), tolerability (occurrence and severity of side effects), and safety (adverse events) of tsDCS and textured shoe insoles when combined with a complex terrain walking task in older adults. The second goal was to examine preliminary efficacy for increasing locomotor learning, as defined by retention of gains in walking speed between a baseline visit of task practice, and a subsequent follow-up visit. Changes in executive control of walking (prefrontal cortical activity) between the baseline and follow-up visits was also examined. The study had two hypotheses: (1) the interventions would be safe and well-tolerated by participants, and (2) compared to a control group with smooth insoles and sham tsDCS, effect sizes would support better retention of task performance in the groups who received active tsDCS, or textured insoles, or a combination of both.

## Materials and Methods

### Overview

Study participants were screened for enrollment criteria, and those who met criteria attended two study visits. The study used a parallel groups design, in which each participant was randomly assigned to one adjuvant group (out of four) receiving active or sham tsDCS, plus textured or smooth shoe insoles. At the baseline visit, participants completed 15 trials of a complex terrain walking task involving stepping over foam obstacles and walking on compliant surfaces. They simultaneously received the tsDCS (active or sham) and insole (smooth or textured) adjuvants based on group assignment. At the follow-up session, participants completed three trials of the complex terrain walking task with no adjuvants. The primary outcome measure was walking speed with a correction applied for number of obstacle strikes, to account for both speed and accuracy. To assess use of executive control resources during walking, prefrontal cortical activity was measured with functional near infrared spectroscopy (fNIRS).

### Recruitment of Older Adult Participants

Recruitment was conducted by mailing advertising flyers to patients of the North Florida/South Georgia Veterans Health System using a mailing list generated by the VA Informatics and Computing Infrastructure. The mailing list included individuals whose medical record indicated age 65 or greater, primary residence in a local zip code, and absence of major medical conditions (i.e., absence of ICD-9/ICD-10 codes for major diseases of the nervous system, circulatory system, musculoskeletal system, etc.). A standardized screening questionnaire was used to interview individuals by telephone. Inclusion criteria included self-reported difficulty with walking, climbing stairs, or doing daily chores. Exclusion criteria included diagnosed neurological disorder/injury; severe arthritis in lower extremities (such as awaiting joint replacement); major cardiac, vascular, pulmonary, or renal disease; cancer treatment in the past year (other than for early stage skin, breast, or prostate cancers); diagnosis of a psychological condition (e.g., schizophrenia, bipolar disorder); bone fracture or musculoskeletal surgical procedure within prior 6 months; contra-indications to lumbar spinal electrical stimulation (e.g., low back pain, prior spinal surgical procedure); pacemaker or other electronic medical device; severe obesity; current use of prescription medications affecting the central nervous system; and current participation in physical therapy.

### Onsite Screening

All onsite study procedures were conducted at a research center in an outpatient hospital setting. Participants provided written informed consent upon arriving to the research center. The aforementioned inclusion and exclusion criteria from the phone screening were reviewed again to ensure consistency, and additional screening assessments were conducted. Preferred 10-meter walking speed was measured, with the goal of enrolling participants who had mild to moderate mobility deficits [walking speed < 1.0 meters/second (Studenski, 2009)]. Body mass index (BMI) was calculated, and people with excessively high BMI (>30) were excluded due to the likelihood that body fat would reduce the amount of tsDCS current reaching the spinal cord. Tactile somatosensation was measured using two-point discrimination on the sole of each foot, and people with poor sensation were excluded [discrimination poorer than 20 mm, which is consistent with scores from people with diabetic neuropathy (Periyasamy et al., 2008)]. Some minor exceptions were made to these criteria to facilitate sufficient enrollment of participants (e.g., people who had walking speed slightly above 1.0 m/s, BMI slightly above 30, or similar minor exceptions). Participants were also excluded if they used medications known to affect the central nervous system (particularly those acting as sodium channel blockers or affecting the neurotransmitters glutamate or gamma-aminobutyric acid) due to potential influence on neuroplasticity and motor learning. Several additional assessments were conducted to further characterize the participants. These included fastest safe 10-meter walking speed, skinfold thickness (abdominal, suprailiac, and subscapular sites), vibratory somatosensation on the sole of the foot, Trail Making Test of cognitive function (Tombaugh, 2004), and Activities Specific Balance Confidence Scale (Powell and Myers, 1995).

### Study Design

Following screening, participants were stratified by sex and then randomly assigned to receive either active or sham tsDCS (1:1 allocation), and either textured or smooth shoe insoles (1:1 allocation). Randomization was accomplished by following an assignment sequence that was generated by the Principal Investigator at the beginning of the study. Block randomization was used to ensure that samples sizes remained approximately equal across groups. This yielded four parallel groups: sham tsDCS and smooth insoles (*sham/smooth*; control group), sham tsDCS and textured insoles (*sham/textured*), active tsDCS and smooth insoles (*active/smooth*), and active tsDCS and textured insoles (*active/textured*). Participants were blinded to tsDCS assignment based on the characteristics of the sham procedure, as described below. Research staff were not blinded to group assignment, because the tsDCS stimulator setting and type of insoles were readily visible to the staff members who ran the session. This study was registered with ClinicalTrials.gov (NCT03667573). Enrollment was open between December 2018 and August 2021. Participant recruitment was stopped at the end of the grant funding period.

### Complex Terrain Walking Task and Locomotor Learning

At the baseline visit, participants performed 15 practice/learning trials over the complex terrain course. A new trial began every 2 min (e.g., trial 1 at 2 min, trial 2 at 4 min, and trial 15 at 30 min). To minimize fatigue, participants sat on a bench immediately after completing each trial. At the follow-up visit, all walking procedures were identical but there were only three trials over the complex terrain course.

**FIGURE 1:**
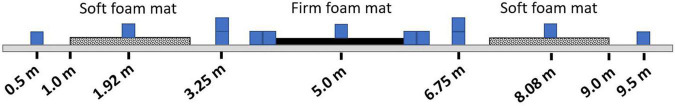
Complex terrain walking course. Participants walked over a 10-meter course consisting of nine foam obstacles and three foam mats. This illustration shows terrain placement, but the exact dimensions of the obstacles and mats are not drawn to scale.

The complex terrain task involved walking across a 10-meter course consisting of nine obstacles and three sections of compliant (soft) walking surface (Figure 1). The dimensions of a single obstacle were 10 × 61 × 10 cm (length x width x height). For safety, the obstacles were made from a very soft foam that would compress easily if stepped on. The compliant surfaces were foam exercise mats, with dimensions of 183 × 61 × 5 cm. Two of the mats were soft and one was firm. The front and rear edges of the mats were taped to the floor to prevent sliding. The layout was designed to be symmetrical around the center point, so participants would encounter the same terrain in the same order when starting from either end of the course. The participants walked an additional meter at the beginning and end of the complex terrain 10-meter course to mitigate the effect of acceleration and deceleration on walking speed measurements.

Participants were instructed to walk across the course at their fastest safe speed and to avoid obstacle strikes. The instruction to walk at fastest safe speed was repeated before every trial. A stopwatch was used to measure the time taken to complete each pass over the complex terrain course. The number of obstacle strikes occurring for each trial was also counted. Study staff repositioned any obstacles that were struck during the walking trials. No other specific instructions were provided. The use of fast walking speed increased the challenge of the task, and also helped to ensure that participants would not select widely varying strategies of task performance (e.g., deliberately walking at a slow speed). All participants wore a gait belt around their waist, and a research staff member was nearby to provide assistance if a participant became unsteady. The research personnel did not provide physical assistance or verbal guidance unless necessary for safety.

The primary performance outcome measure on the complex terrain task was “corrected speed,” which is walking speed (in meters/second) multiplied by the percentage of obstacles that were crossed successfully (i.e., without striking the obstacle). Using this approach, the penalty for each obstacle strike is an 11.1% reduction in the corrected speed value.

### Motion Analysis Assessment for the Complex Terrain Task

Kinematic data were acquired by attaching reflective markers to anatomical landmarks on the head, trunk, arms, legs and feet using the Helen Hayes configuration (Kadaba et al., 1990) modified with additional marker triads attached to rigid plates located on each shank and thigh segment (Allen et al., 2011). The 3-dimensional location of each marker was sampled at 100 Hz using an 18 camera motion capture system and data acquisition system (Vicon Motion Systems, CO, United States). The kinematic data were low-pass filtered using a fourth-order Butterworth filter with cutoff frequency of 6 Hz. An 8-segment model (Visual 3D software, C-Motion, Inc., Germantown, MD, United States) was used to calculate body center-of-mass position. Using the established lab coordinate system, the course was oriented such that each subject’s anterior/posterior axis coincided with the forward progression of the obstacle course. The mediolateral trajectory of the center of mass was normalized (detrended) by subtracting the value of its best-fit linear regression. The standard deviation of the mediolateral position of the center of mass was then calculated for each walking trial.

### Transcutaneous Spinal Cord Direct Current Stimulation

Transcutaneous spinal direct current stimulation was set up and administered at the baseline visit only, using a commercially available stimulator (1 × 1 tES Clinical Trials Stimulator, Soterix Medical Systems, New York, NY, United States). Stimulation was delivered simultaneously with the 15 trials on the complex terrain course. The stimulator is lightweight and was placed in a backpack made of clear plastic which was worn by the participant. The clear plastic allowed research personnel to view an electrode contact quality indicator on the stimulator to confirm that stimulation quality remained high throughout the session. The anode lead was connected to a carbon rubber electrode (4.5 × 4.5 cm) embedded within a thin 5 × 10 cm sponge (EasyPad, Soterix Medical Systems, New York, NY, United States). The sponge was evenly moistened on the front and back sides with 5.7 mL of 0.9% saline solution (11.4 mL total per sponge). The anode electrode was oriented vertically and centered over the 11th and 12th thoracic spinal processes, which overlays the lumbar region of the spinal cord. Two cathode leads were each connected to separate carbon rubber electrodes embedded within a thin 5 × 7 cm sponge. The sponge was evenly moistened on the front and back sides with 4 mL of 0.9% saline solution (8 mL total per sponge). The two cathode electrodes were placed on each side of the umbilicus in approximately the same horizontal plane as the anode, with a lateral distance of 5 cm between the center of the umbilicus and the center of each sponge. This electrode configuration has been shown to appropriately target the lumbosacral spinal cord, as validated by modeling of electrical current flow (Parazzini et al., 2014) and by gains in lower extremity motor performance (Berry et al., 2017). For participants assigned to active tsDCS, 30 continuous minutes of 2.5 mA stimulation was delivered. An identical electrode montage was used for the sham condition, except the stimulation was ramped up to 2.5 mA over a period of 30 s, held constant for 30 s, and then ramped back down over 30 s to 0 mA. The stimulator then delivered no current for 27 min, followed by another ramp up, hold, and ramp down. This sham approach is widely used in the field of direct current stimulation, and has been shown (in studies of transcranial DCS) to be an effective sham procedure that gives the sensation of active stimulation but without delivering a meaningful dose (Woods et al., 2016).

### Shoe Insoles for Somatosensory Feedback

**FIGURE 2:**
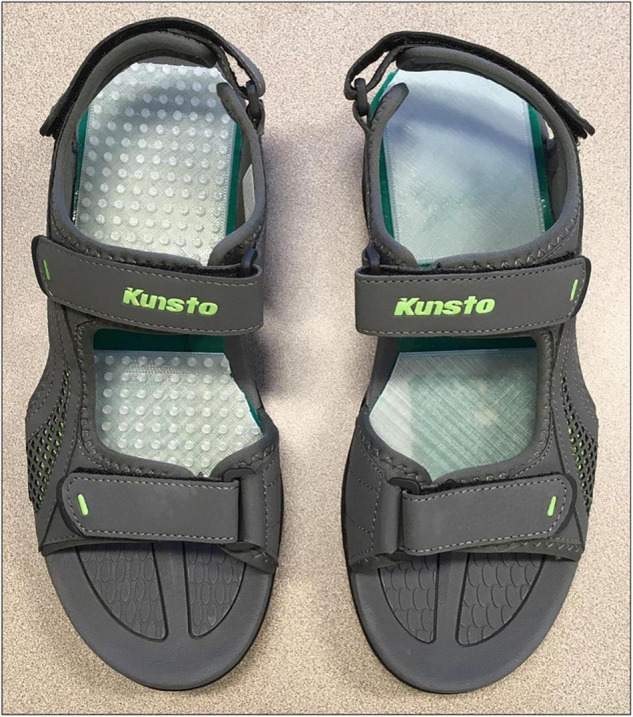
Insole intervention. Standardized walking sandals were worn by all participants. At baseline, participants were randomly assigned to either textured insoles (example on left) or smooth insoles (example on right). At follow-up, participants wore the walking sandals without insoles.

At baseline and follow-up visits, footwear was standardized by having participants wear a pair of adjustable open-toe walking sandals (Figure 2, Kunsto Sports Sandal). The lab stocked multiple sizes of sandals to accommodate all participants, and a snug fit was ensured with adjustable hook-and-loop straps across the forefoot, midfoot, and behind the heel. The sandals facilitated correct placement of the insoles. Participants were also provided with a new pair of cotton crew socks at each session, to standardize the interface between the footwear and the sole of the foot.

At the baseline visit only, participants were randomly assigned to either textured insoles or smooth insoles. At the follow-up visit, no insoles were used. The insoles were designed in CAD software (SolidWorks, Dassault Systemes, Vélizy-Villacoublay, France) and printed with a 3D printer (Lulzbot Taz 6, Fargo Additive Manufacturing Equipment 3D, LLC, Fargo, ND, United States) using plastic material (polylactic acid). The smooth insoles were cut from a flat piece of 1.5 mm thickness. The material is sufficiently flexible to accommodate natural bending of the foot throughout the stance phase of walking. The textured insoles used the same base material, but also had raised cylindrical bumps (2.5 mm height with flat top surface, and 1.9 mm diameter) spaced 1 cm apart in a grid pattern. The insoles were cut into three sections (front, middle, and rear), and the middle section was discarded. This allowed placement of the front and rear sections to be customized for proper fit within the sandal, and correct placement of the insole under the heel and forefoot (metatarsal heads but not the toes; Figure 2). To prevent the insoles from shifting position within the sandal, they were mounted to a non-slip material (Non-Slip Reel, Dycem Corporation, Smithfield, RI, United States). The participants were not blinded to insole assignment, though study staff refrained from discussing details of the study design or hypothesis with participants.

### Intervention Feasibility, Tolerability, and Safety

Feasibility refers to participant enrollment and drop-outs, and was quantified based on the number of participants completing phone screening, onsite screening visit, baseline visit, and follow-up visit.

Tolerability refers to side effects reported due to tsDCS or textured insoles. Given the generalized delivery of stimulation to the lumbar region of the spinal cord, we interviewed participants about potential motor, sensory, and autonomic side effects. Participants used an 11-point rating scale where 0 represents “none” and 10 represents “strongest/worse possible.” For tsDCS, the following items were rated: tingling, itching, burning, pain, fatigue, nervousness, headache, muscle spasms, mood change, urinary urgency, abdominal/pelvic sensations, and sweating. For insoles, the same rating scale was used to rate pain on the sole of the foot. Questionnaires were administered at each study visit. At the baseline visit, the ratings were obtained prior to the intervention, immediately after the intervention (in reference to “during the intervention”), and following a 10-min seated rest period after the walking intervention concluded. At the beginning of the follow-up visit, the same set of ratings was obtained but in reference to “since your last study visit.”

Safety refers to the occurrence of adverse events, based on guidelines from the National Institutes of Health. An adverse event is any untoward or unfavorable medical occurrence in a human study participant, including any abnormal sign, symptom, or disease, temporally associated with the participant’s involvement in the research, whether or not considered related to participation in the research.

### Prefrontal Brain Activity Measured With Functional Near Infrared Spectroscopy

**FIGURE 3:**
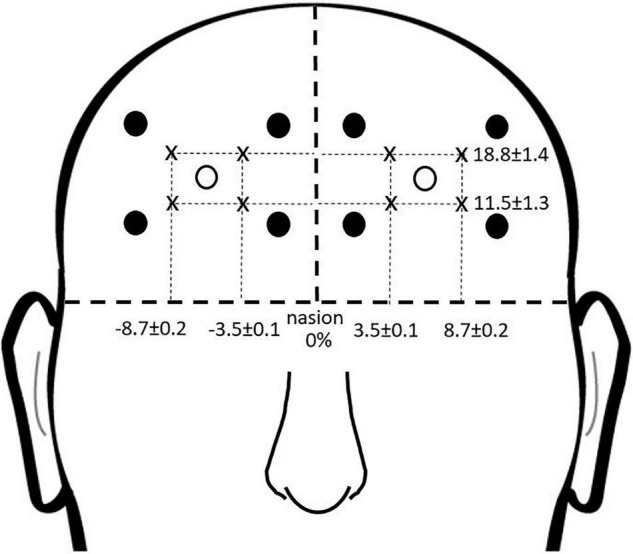
fNIRS recording sites. Group mean fNIRS recording sites expressed as a percentage of head circumference (lateral direction) and nasion to inion distance (vertical direction). Light emitters are shown as closed circles and light detectors as open circles. The recording site is estimated as halfway between each emitter-detector pair, and shown with an “x.”

Prefrontal activity was measured during the complex terrain task with continuous-wave functional near infrared spectroscopy (fNIRS; OctaMon, Artinis Medical Systems, Nijmegen, Netherlands) (Menant et al., 2020). Participants wore a headband with eight embedded light sources that emitted near infrared light at wavelengths of 760 and 850 nm, along with two near infrared light detectors. This yielded eight fNIRS channels, which were distinguished by time division multiplexing. Each channel was recorded at 10 Hz. The bottom of the headband was positioned just above the eyebrows, and the middle of the headband was aligned with the midline of the face. The source-detector optode locations on the headband were fixed, and were separated by a distance of 3.5 cm. To report estimated anatomical recording sites for each channel, we measured the mid-point location between each light emitter-detector pair and report this location in reference to the International 10-10 System (Herold et al., 2017). Lateral placement was measured in the transverse plane as a percentage of head circumference. Vertical placement was measured in the sagittal plane as percentage of the nasion to inion distance. The lateral and vertical recording sites relative to the nasion are shown in Figure 3. The lower medial optodes were located at approximately FP1 and FP2 for left and right sides, respectively. The lower lateral optodes were located at approximately AF7 and AF8 for left and right sides, respectively. The upper medial optodes were located at approximately AF3 and AF4 for left and right sides, respectively. These measurement locations correspond to sub regions of Brodmann Area 9 (upper channels) and Brodmann Area 10 (lower channels) (Koessler et al., 2009).

Functional near infrared spectroscopy was measured using a block design where active walking periods were compared with reference periods. Immediately after completing each walking trial, participants sat on a bench for about 60–70 s. They were then asked to stand up, and after several seconds were instructed to count slowly (about one number per second) for the remainder of the rest period (Holtzer et al., 2015; Chen et al., 2017). This low-demand task during the reference period was used to help prevent mind wandering and enhance consistency across participants (Herold et al., 2018). Prefrontal oxygenated hemoglobin (O2Hb) concentrations were calculated according to the modified Beer-Lambert law with differential path length factor of 6 (Herold et al., 2017), then analyzed with custom programs in Matlab (MathWorks, Natick, MA, United States). Preprocessing of the fNIRS signals included a low-pass filter with cutoff frequency at 0.14 Hz to reduce physiological noise (Huppert et al., 2009; Holtzer et al., 2011). fNIRS is based on blood oxygen level dependent responses, which have a time lag of several seconds (Herold et al., 2018). The duration of each walking trial was too short to allow the signal amplitude to plateau, so the value used for analysis was the peak amplitude occurring between the starting point of the trial and within 5 s after the trial ended (a period of time which should reflect prefrontal activity occurring during the walking task). For each reference period, the value used was the minimum value recorded. Task-related change in prefrontal O2Hb (ΔO2Hb) was calculated using the formula: Δ*O2Hb* = *Active O2Hb – Reference O2Hb*. The ΔO2Hb value was calculated separately for each fNIRS channel and walking trial, using the reference period that immediately preceded each trial. All eight fNIRS channels were averaged together for a composite measure of prefrontal activity, as the small sample size of this study was insufficient for investigating differences across channels.

### Data Analysis and Statistics

Differences between groups on demographic, clinical, and mobility function measures (Table 1) were assessed with ANOVA models. To assess the effect of the tsDCS and insole interventions on corrected walking speed, center of mass mediolateral variability, and prefrontal brain activity during the complex terrain task, we calculated the change between baseline and follow-up visits. The data were first averaged across all trials for each session, to obtain a baseline mean and a follow-up mean for each participant. Then the difference was calculated between baseline and follow-up sessions for each participant. These difference scores were used to calculate group means and effect sizes (Cohen’s d).

**TABLE 1:** Participant demographics, clinical assessments, and walking function.

	Sham tsDCS/Smooth insoles	Sham tsDCS/Textured insoles	Active tsDCS/Smooth insoles	Active tsDCS/Textured insoles	Full sample
Sample size	*n* = 7	*n* = 5	*n* = 5	*n* = 6	*n* = 23
Male/Female	5/2	5/0	4/1	5/1	19/4
Age (years)	79.3 ± 7.5	77.2 ± 9.9	75.8 ± 5.8	78 ± 7.7	77.7 ± 7.4
Preferred walking speed (m/s)	0.97 ± 0.25	0.96 ± 0.18	0.95 ± 0.18	1.04 ± 0.14	0.98 ± 0.18
Fastest walking speed (m/s)	1.4 ± 0.36	1.39 ± 0.4	1.29 ± 0.37	1.48 ± 0.28	1.38 ± 0.34
ABC scale (% confidence)	79 ± 29.6	88.4 ± 12.2	79.8 ± 25.9	81.1 ± 16.8	81.8 ± 21.6
Trail making test (s; Part B – A)	89.1 ± 60.9	31.2 ± 3.3	98.2 ± 88.3	65.6 ± 24.1	72.4 ± 56.6
Vibratory somatosensation (μm)	36.5 ± 19.6	65.5 ± 27.6	40.4 ± 28.9	56.9 ± 36.1	49.6 ± 28.7
Body mass index (kg/m^2^)	24.1 ± 2.2	27.1 ± 2.9	27.2 ± 3.4	27.8 ± 4	26.4 ± 3.3
**Skinfold thickness:**					
abdominal (mm)	24 ± 2.6	23.4 ± 5	20.6 ± 5	19.2 ± 4.4	21.9 ± 4.5
suprailiac (mm)	18.7 ± 2.5	19.2 ± 6	18.8 ± 8.7	18.9 ± 7.6	18.9 ± 6
subscapular (mm)	18.5 ± 4.4	17.6 ± 7.4	21.8 ± 6.3	19.8 ± 9	19.4 ± 6.5

## Results

### Participants

**FIGURE 4:**
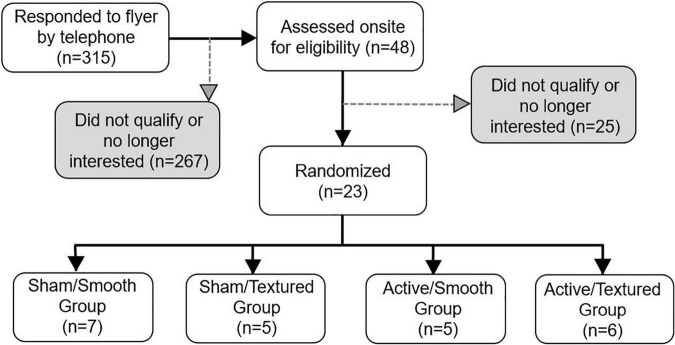
Flow diagram of participant enrollment and randomization. All randomized participants completed the baseline and follow-up visits successfully.

Twenty-three participants were randomized to the intervention, and all completed the full study protocol. There was a higher proportion of male participants due to our focus on recruiting United States military veterans (consistent with funding from the United States Department of Veterans Affairs). Figure 4 shows the flow of participants through each stage of the study. Demographic, clinical, and mobility function data are presented in Table 1. There were no statistically significant differences between groups for any of these measures.

### Feasibility, Tolerability, and Safety

Feasibility was based on participant enrollment and retention. The number of participants randomized to the intervention was low as compared to the total number of individuals who agreed to be screened by telephone (7.3% enrollment rate), and those who subsequently attended an onsite screening visit (47.9% enrollment rate). The most common reasons for exclusion were presence of major or unstable disease, no walking difficulty (we sought participants with self-reported walking difficulties), high body mass index (excess adipose tissue would interfere with tsDCS), and poor somatosensation in the feet (which may interfere with the effect of textured insoles). For participants who were randomized to the intervention, there was a 100% completion rate for the baseline and follow-up visits.

Tolerability was based on side-effects reported due to tsDCS or textured insoles. The side effects reported for tsDCS were negligible or absent for every symptom that was assessed. The group mean score (magnitude of severity) for each symptom was less than 1 point (out of 10) for both the active tsDCS group and sham tsDCS group at every timepoint. Furthermore, group mean differences between the pre-intervention timepoint and during the intervention were less than 1 for every symptom.

**FIGURE 5:**
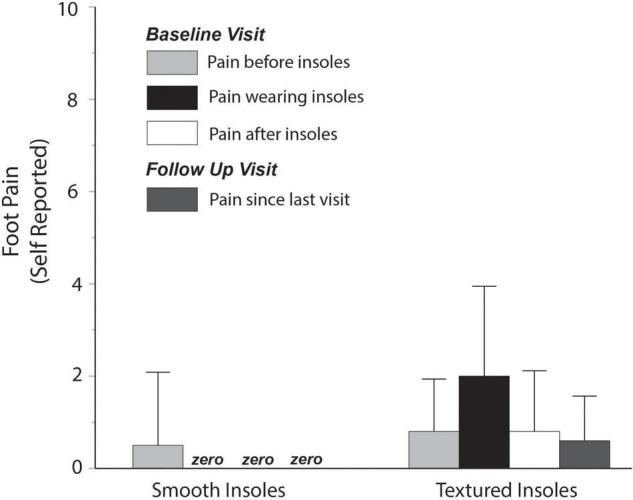
Self-reported foot pain. Pain reported while wearing textured insoles was low, with an average rating of approximately 2 out of 10.

Foot pain related to the insole intervention was also low (Figure 5), with a group mean value of approximately 2 points (out of 10) for participants randomized to textured insoles. One participant rated the pain as a 6. When expressed relative to self-reported pain before the intervention or after the intervention, the increase in pain when wearing the textured insoles was less than 1.5 points, on average.

Safety was based on occurrence of adverse events. There were no adverse events in any study participants, and no participants fell during the study visits.

### Performance on the Complex Terrain Walking Task

**FIGURE 6:**
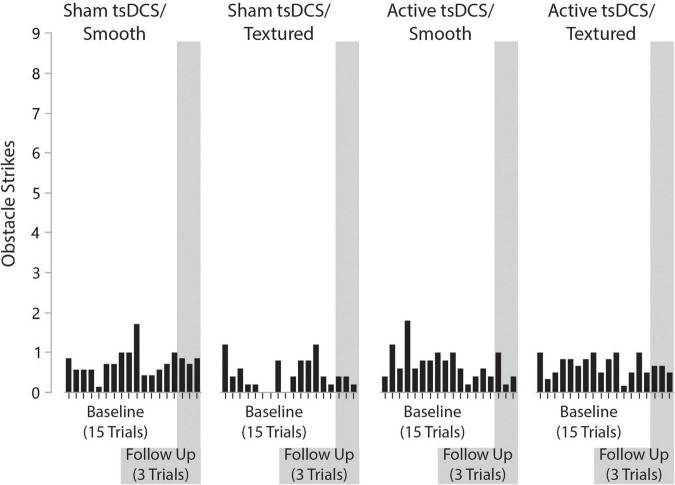
Number of obstacle strikes by group, trial number, and visit. On average, there was approximately one obstacle strike (out of nine obstacles) per walking trial. The number of obstacle strikes across visits was consistent.

**FIGURE 7:**
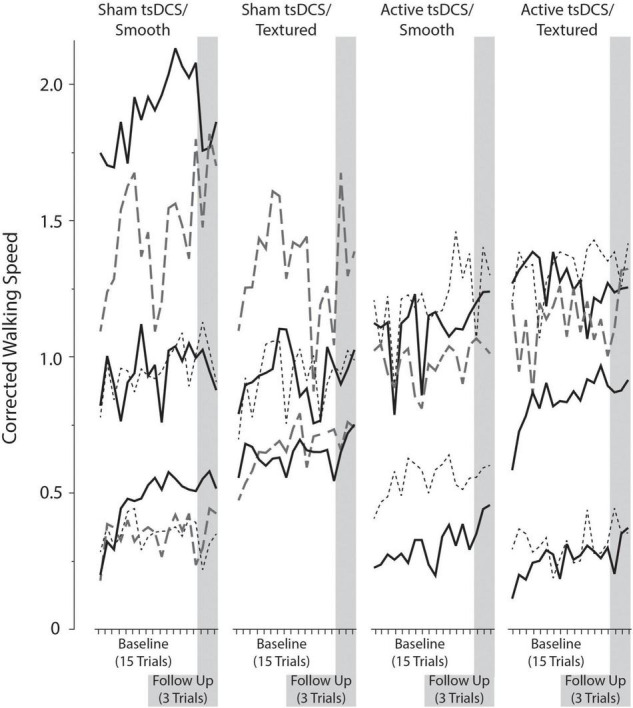
Corrected walking speed across each trial for all study participants.

**FIGURE 8:**
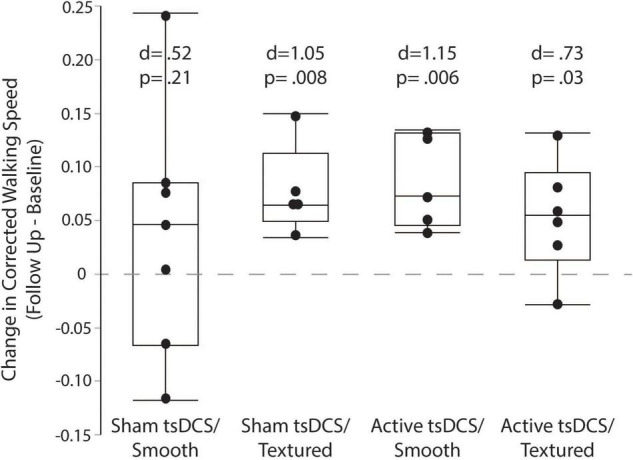
Corrected walking speed. Box plots showing the change in corrected walking speed between the baseline and follow-up visits. For each participant, all trials were averaged within each session before calculating the change as follow-up minus baseline. The groups with active tsDCS and/or textured insoles showed larger and more consistent retention of performance than the control group (sham/smooth).

The primary outcome measure for performance on the complex terrain task was corrected walking speed, which is walking speed (in meters/second) multiplied by the percentage of obstacles that were crossed successfully. The group mean number of obstacle strikes across each of the walking trials during the baseline and follow-up visits are shown in Figure 6. On average, there was approximately 1 obstacle strike (out of 9 obstacles) per walking trial. For each participant, the corrected speed across all trials of both sessions is shown in Figure 7. For each group, the mean and effect size was calculated for the change in corrected walking speed between the baseline and follow-up sessions (calculated as follow-up minus baseline). Box plots (with median and quartile values) are shown in Figure 8. For *sham/smooth* the mean was 0.039 ± 0.12 (*d* = 0.52, *p* = 0.21). In contrast, all three experimental groups showed a statistically significant effect. For *sham/textured* the mean change 0.078 ± 0.04 (*d* = 1.05, *p* = 0.008). For *active/smooth* the mean change was 0.085 ± 0.04 (*d* = 1.15, *p* = 0.006). For *active/textured* the mean change was 0.054 ± 0.054 (*d* = 0.73, *p* = 0.03). The percentage of participants in each group who experienced an increase in walking speed at the follow-up visit relative to baseline was 57% for *sham/smooth*, 100% for *sham/textured*, 100% for *active/smooth*, and 83% for *active/textured*.

A secondary outcome for performance on the complex terrain walking task was mediolateral variability of the center of mass. For each group, the mean and effect size was calculated for the change in mediolateral center of mass variability between the baseline and follow-up sessions (calculated as follow-up minus baseline). Only the *active/smooth* group showed a significant effect, with a mean change of −0.75 ± 0.27 cm (*d* = 2.77, *p* = 0.004). For *sham/smooth* the change was −0.05 ± 0.46 cm (*d* = 0.11, *p* = 0.81). For *sham/textured* the mean change was −0.31 ± 0.57 cm (*d* = 0.54, *p* = 0.30). For *active/textured* the mean change was −0.26 ± 1.1 cm (*d* = 0.24, *p* = 0.63).

### Functional Near Infrared Spectroscopy Prefrontal Brain Activity

**FIGURE 9:**
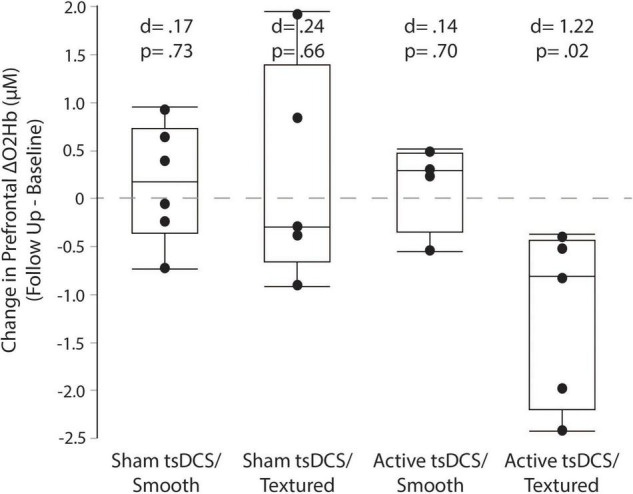
Prefrontal cortical activity measured by fNIRS. Box plots showing the change in prefrontal fNIRS between the baseline and follow-up visits. For each participant, all trials were averaged within each session before calculating the change as follow-up minus baseline. Only the active/textured group showed a significant reduction in prefrontal activity at follow-up. Lower prefrontal activity suggests fewer executive control resources are needed to perform the complex terrain task.

For each group, the mean and effect size were calculated for the change in prefrontal cortex activity between the baseline and follow-up sessions. Box plots (with median and quartile values) are shown in Figure 9. Only the *active/textured* group showed a significant effect, with a mean change of −1.21 ± 0.93 μM (from 2.32 to 1.10 μM, *d* = 1.22, *p* = 0.02). For *sham/smooth* the mean change was 0.17 ± 0.62 μM (from 3.47 to 3.63 μM, *d* = 0.17, *p* = 0.73). For *sham/textured* the mean change was 0.24 ± 1.15 μM (from 2.71 to 2.95 μM, *d* = 0.24, *p* = 0.66). For *active/smooth* the mean change was 0.14 ± 0.47 μM (from 2.06 to 2.20 μM, *d* = 0.14, *p* = 0.70).

## Discussion

The results of this study show that tsDCS and textured insoles are feasible, well-tolerated, and safe intervention adjuvants when combined with a single locomotor learning session of walking on complex terrain in older adults. Furthermore, preliminary effect size data warrant future studies to assess the efficacy of tsDCS and textured insoles for improving complex walking performance when combined with short-term locomotor learning and/or longer term rehabilitation interventions.

### Feasibility, Tolerability, and Safety

The feasibility of recruiting and enrolling participants was affected by exclusion criteria specific to each intervention adjuvant. Specifically, people with high body mass index were excluded because of concern that adipose tissue beneath the tsDCS electrodes would reduce the amount of current reaching the spinal cord. People with poor somatosensation in the feet were excluded because sensory feedback from the textured insoles may be less effective if cutaneous tactile receptors are not able to robustly transmit afferent information through the peripheral nerves. Both obesity and peripheral neuropathy are common in older adults, and contributed to the number of participants who screened out of the study. The recruitment yield would likely be better for studies that test tsDCS or insoles alone, rather than in combination. Given that combination therapies are becoming increasingly popular, there is a need to acknowledge that some may only be appropriate for a segment of the patient population. For participants who met enrollment criteria and were randomized to the intervention, the feasibility of completing both the baseline and follow-up visits was excellent (100% completion rate).

The tsDCS and insole interventions were found to be well-tolerated by the participants. For tsDCS there were reports of very mild tingling/burning sensation at the electrode sites (average rating less than 1 out of 10). All other potential side effects of tsDCS were negligible or completely absent. The textured insoles were generally comfortable for participants, with the exception of one person who reported a rating of 6 out of 10 for pain. The safety of the intervention was excellent, based on no reported adverse events.

It is important to consider that our intervention was only 30 min long for a single session, so the exposure to tsDCS and insoles was brief. We are unsure if prolonged and/or daily use would lead to issues with tolerability. Another potential issue is that neural habituation might moderate the effects of tsDCS and/or textured insoles when used during a longer session, or for multiple sessions. The feasibility and safety of these intervention approaches over longer time periods will be an important area for future research.

### Performance on the Complex Terrain Walking Task

There was considerable variability in performance on the complex terrain task for both absolute walking speed and amount of locomotor learning. A possible explanation for this variability, particularly within the baseline visit, is that participants might have experimented with different performance strategies during different walking trials. While we always instructed participants to walk at their fastest safe speed, we did not provide any additional instructions because we wanted participants to determine their preferred control strategy for the complex terrain task.

Although the small sample size of this study is insufficient to draw definitive conclusions about efficacy, it was encouraging that between-session retention (or gains) in task performance had larger effect sizes for the groups with active tsDCS and/or textured insoles. The mean change in corrected walking speed (follow up minus baseline) was approximately two-fold higher for both the *active/smooth* and *sham/textured* groups relative to the *sham/smooth* control group. The within-group effect sizes for both of these experimental groups were large (*d* > 1.0) and statistically significant, versus the control group’s moderate effect size (*d* = 0.52). We had expected that the active/textured group would show the largest benefit to retention of performance because of the combined intervention approach. While the effect size was somewhat larger than the sham/smooth control group (*d* = 0.73), it remained below the level of the other two experimental groups.

Like walking speed, the reduction in center of mass mediolateral variability during the complex terrain task also showed trends favoring the groups with active tsDCS and/or textured insoles. Reduction in center of mass mediolateral variability may imply better stability during task performance. Compared to the *sham/smooth* group, the reduction in variability for the other three groups ranged from being 5–15 times more pronounced.

### Functional Near Infrared Spectroscopy Prefrontal Brain Activity During the Complex Terrain Walking Task

This study examined whether the tsDCS and textured insole adjuvants would help to reduce prefrontal activity at the follow-up session relative to baseline. Reduced prefrontal activity at follow-up might indicate better walking automaticity, suggesting improved spinal control (Clark, 2015), thereby offloading the demand for executive control of walking (Clark et al., 2014; Clark, 2015). It could also indicate more efficient cognitive processing in the cortex (Reuter-Lorenz and Cappell, 2008; Chen et al., 2017; Chatterjee et al., 2020). Our results show a substantial reduction in prefrontal activity for the *active/textured* group, but not for any of the other groups. This finding could mean that the combination of active tsDCS and textured insoles was the most potent intervention for promoting neuroplasticity and changes in the neural control strategy. However, if true, this change in neural control did not directly translate to better walking performance for the active/textured group relative to the groups receiving just active tsDCS or just textured insoles. Larger sample sizes are needed to confirm the fNIRS findings, and to better understand the relationship between neural control strategies and behavioral measures of locomotor learning.

### Considerations for Transcutaneous Spinal Cord Direct Current Stimulation

Numerous prior studies have demonstrated the ability of tsDCS to modify spinal reflex activity and/or behavioral outcomes (Cogiamanian et al., 2008; Winkler et al., 2010; Lamy et al., 2012; Hubli et al., 2013; Bocci et al., 2015; Berry et al., 2017). Unlike most prior studies of tsDCS which delivered the stimulation while participants were at rest, we delivered the stimulation simultaneously with performance of the complex terrain walking task. Our intention was to promote excitation in spinal circuits, in order to create a neurophysiological environment that is conducive to activity-dependent neuroplasticity. We placed the anode electrode over the lumbar spinal cord, because prior studies have reported that the anode produces hypopolarization of neuron cell membranes (Woods et al., 2016). This excitation from tsDCS may increase the probability of action potential firing in neurons that also receive task-specific excitatory potentials. This goal of reinforcing task-specific neural activity may create an opportunity for strengthening synapses through Hebbian neuroplasticity (Kronberg et al., 2020).

Transcutaneous spinal direct current stimulation might promote learning of the complex terrain walking task by acting on multiple neural control mechanisms. These include transmission of descending motor commands through the spinal cord to adapt leg movement during obstacle crossing (Haefeli et al., 2011); transmission of peripheral somatosensory feedback through the spinal cord (Frigon and Rossignol, 2006; Frigon, 2017) (which may act on spinal or supraspinal circuits); and/or by influencing activity in the CPG that controls intermuscular coordination during walking (Dietz, 2003; McCrea and Rybak, 2008; Gosgnach et al., 2017). While the conceptual objectives of tsDCS are relatively straightforward, we acknowledge that the neurophysiological effects are very complex. Delivery of electrical current to the spinal cord with tsDCS is imprecise, and might influence both intended and unintended targets. Future research will be necessary to better understand the effects of tsDCS on spinal circuits and on locomotor learning, including implications of different electrode configurations and polarities.

### Considerations for Somatosensory Feedback

Somatosensory information is known to be crucial for motor learning (Yen et al., 2014; Bernardi et al., 2015). Our use of textured shoe insoles was intended to increase afferent feedback pertaining to gait events, such as more distinct signaling of heel strike, gradations in weight bearing, and timing of terminal stance. This objective is based on prior research showing that afferent input from the lower extremity plays an important role in timing of the locomotor CPG (Frigon and Rossignol, 2006; Guertin, 2009; Guertin, 2012). Load receptors detect weight bearing at heel strike and initiate activity in extensors during the stance phase, as well as activate muscles that generate forward propulsion (Hiebert and Pearson, 1999; Pearson, 2008). Hip extension and reduced weight bearing in late stance provide a signal to increase flexor activity and initiate swing phase (Hiebert et al., 1996; McVea et al., 2005). Somatosensory information can also reset the extension (stance) or flexion (swing) phase of the CPG when gait cycles are disrupted by perturbations (Hiebert et al., 1996; Schomburg et al., 1998), such as with altered compliance of the walking surface or due to tripping (Yamasaki et al., 2003). By increasing the amount of tactile somatosensation during practice of the complex terrain walking task (particularly when walking on compliant surfaces), the CPG circuits may learn a more flexible repertoire of timings to accommodate non-uniform walking surfaces. If so, this may improve spinal control, and thereby reduce reliance on executive control resources to coordinate walking. When combined with tsDCS, this effect might be further enhanced as suggested by the reduced prefrontal activity observed at follow-up in the *active/textured* group.

Our attention to somatosensory feedback is also consistent with a substantial body of evidence showing cross-sectional associations between better somatosensory function (i.e., perception of tactile and vibratory sensation on the foot) and better performance on various measures of walking and balance (Resnick et al., 2000; Mold et al., 2004; Deshpande et al., 2008; Cruz-Almeida et al., 2014). Furthermore, prior studies have shown immediate improvements in measures of walking and balance performance when participants wear vibrating or textured insoles (Priplata et al., 2003; Palluel et al., 2008, 2009; Qiu et al., 2012). Future studies are warranted to better understand how augmented somatosensory feedback might enhance rehabilitation outcomes.

### Study Limitations

There are limitations to this study that are important to acknowledge. Our conceptual framework for this study proposed excitation to spinal circuits, including the locomotor CPG, as a mechanism for how tsDCS and textured insoles might benefit locomotor learning. However, potential mechanisms not measured in this study may also be involved, such as reflex circuits or supraspinal circuits of sensorimotor control. Another factor to consider is that our study participants had to meet a fairly restrictive set of enrollment criteria. Applying these criteria was prudent for the current stage of the research, but limits generalizability. This study also had a small sample size in each group, and was not designed to establish the efficacy of each intervention. Likewise, efficacy cannot be easily interpreted because the study was not designed to balance the groups at baseline for any performance measure or demographic measure (other than sex). We also cannot be sure that the tsDCS parameters (duration, intensity, electrode placement, etc.) were ideal. Larger clinical trials with more participants will be needed to address these issues. The results shown here provide justification for pursuing future clinical trials.

## Conclusion

The results of this study show feasibility, tolerability, safety, and preliminary positive outcomes for using tsDCS and/or textured insoles to increase locomotor learning during a complex terrain walking task. These findings provide justification and preliminary evidence for pursuing future larger studies that can more definitively test these interventions in larger samples, and with extended use.

## Data Availability Statement

The raw data supporting the conclusions of this article will be made available by the authors, without undue reservation.

## Ethics Statement

The studies involving human participants were reviewed and approved by the University of Florida Institutional Review Board and the Human Research Protections Program at Malcom Randall VA Medical Center. The patients/participants provided their written informed consent to participate in this study.

## Author Contributions

DC, DF, and EF obtained funding for this project. DC, KH, SW, BC, SP, and JM collected the data. DC, SP, and JM analyzed the data. DC led the manuscript preparation, with contributions from all co-authors (particularly KH, DF, and EF). All authors approved the content of this article.

## Author Disclaimer

The contents of this article do not represent the views of the United States Department of Veterans Affairs or the United States Government.

## Conflict of Interest

The authors declare that the research was conducted in the absence of any commercial or financial relationships that could be construed as a potential conflict of interest.

## Publisher’s Note

All claims expressed in this article are solely those of the authors and do not necessarily represent those of their affiliated organizations, or those of the publisher, the editors and the reviewers. Any product that may be evaluated in this article, or claim that may be made by its manufacturer, is not guaranteed or endorsed by the publisher.
